# Emergence delirium in children is not related to intraoperative burst suppression – prospective, observational electrography study

**DOI:** 10.1186/s12871-019-0819-2

**Published:** 2019-08-08

**Authors:** Susanne Koch, Anna-Maria Stegherr, Leopold Rupp, Jochen Kruppa, Christine Prager, Sylvia Kramer, Astrid Fahlenkamp, Claudia Spies

**Affiliations:** 1 Department of Anaesthesiology and Intensive Care Medicine, Charité-Universitätsmedizin Berlin, Campus Virchow-Klinikum and Campus Charité Mitte, Augustenburger Platz 1, 13353 Berlin, Germany; 2 Charité-Universitätsmedizin BerlinInstitute of Biometry and Clinical Epidemiology, Campus Charité Mitte, Charitéplatz 1, 10117 Berlin, Germany; 3 Department of Paediatria and Neurology, Charité-Universitätsmedizin Berlin, Campus Virchow-Klinikum and Campus Charité Mitte, Augustenburger Platz 1, 13353 Berlin, Germany; 4 Berlin Institute of Health (BIH), Anna-Louisa-Karsch 2, 10178 Berlin, Germany

**Keywords:** Burst suppression, Paediatrics: pre-operative anxiety, EEG: high dose opiates

## Abstract

**Background:**

Emergence-delirium is the most frequent brain dysfunction in children recovering from general anaesthesia, though the pathophysiological background remains unclear. The presented study analysed an association between emergence delirium and intraoperative Burst Suppression activity in the electroencephalogram, a period of very deep hypnosis during general anaesthesia.

**Methods:**

In this prospective, observational cohort study at the Charité - university hospital in Berlin / Germany children aged 0.5 to 8 years, undergoing planned surgery, were included between September 2015 and February 2017. Intraoperative bi-frontal electroencephalograms were recorded. Occurrence and duration of Burst Suppression periods were visually analysed. Emergence delirium was assessed using the Pediatric Assessment of Emergence Delirium Score.

**Results:**

From 97 children being analysed within this study, 40 children developed emergence delirium, and 57 children did not. Overall 52% of the children displayed intraoperative Burst Suppression periods; however, occurrence and duration of Burst Suppression (Emergence delirium group 55% / 261 + 462 s vs. Non-emergence delirium group 49% / 318 + 531 s) did not differ significantly between both groups.

**Conclusions:**

Our data reveal no correlation between the occurrence and duration of intraoperative Burst Suppression activity and the incidence of emergence delirium. Burst Suppression occurrence is frequent; however, it does not seem to have an unfavourable impact on cerebral function at emergence from general anaesthesia in children.

**Trail registration:**

NCT02481999, June 25, 2015.

**Electronic supplementary material:**

The online version of this article (10.1186/s12871-019-0819-2) contains supplementary material, which is available to authorized users.

## Background

Postoperative delirium is the most frequent brain dysfunction in patients recovering from general anaesthesia, mainly seen in preschool children as well as in elderly patients [1, 2].

In preschool children, postoperative delirium occurs during emergence from anaesthesia during the stay in the post-anaesthesiological care unit (emergence delirium) and presents with acute disorientation, crying, agitation and missing response to the surroundings [3]. Emergence delirium (ED) in children is mostly self-limited and has a benign course. However, it increases the risk of self-injury and induces stress to the medical staff as well as to the caregivers. The implications on long-term outcomes especially regarding cognitive function in children is still under discussion.

Pediatric anaesthesiologists are concerned, since it has been proposed that anaesthetic agents may be neurotoxic to the developing brain [4, 5].

Elevated concentrations of the anaesthetic agent sevoflurane cause an increase of epileptiform discharges during anaesthesia induction in children [6]. In a former EEG analysis - focusing on the period of anaesthesia induction in the same patient cohort that is presented here - we have shown that the occurrence of interictal, epileptiform discharges is positively related to the development of ED [7]. In a study from Martin and colleagues analysing multichannel EEG characteristics in 12 children, from whom 5 developed ED, ED was associated with arousal from an indeterminate state (low voltage / fast frequency EEG activity) and an increased frontal cortical functional connectivity [8]. All these EEG characteristics - interictal, epileptiform discharges, indeterminate state, and increased frontal cortical functional connectivity - are related to a cortical state of hyperexcitability.

On the other hand, elevated concentrations of anaesthetic agents will induce a deeper levels of sedation and periods of Burst Suppression in the EEG during general anaesthesia. It has been proven that periods of Burst Suppression and a low index level of anaesthesia trigger postoperative delirium in elderly patients [9–12]. In children, however, two studies analyzing the index level of anaesthesia and its relationship with the occurrence of ED did not find this correlation [13, 14]. A study focusing on intraoperative Burst Suppression periods and the correlation with ED in children is still lacking.

The aim of this study is to analyse if the occurrence and duration of Burst Suppression patterns during general anaesthesia are related to the development of ED in children.

## Methods

This prospective, observational cohort study (NCT02481999) was approved by our local ethics committee on 12th March 2015 (Ethics committee Charité, University Medicine of Berlin / Chairperson Prof. Dr. R. Seeland / EA2/027/15). Written informed consent of either parents or legal proxies was obtained according to the Declaration of Helsinki. We included children aged 0.5–8 years, undergoing elective surgery with a planned duration of > 60 min at our University hospital – Charité / Berlin. Between September 8, 2015 and February 22, 2017 parents or legal proxies of the child were approached by study staff members during stay in the preoperative evaluation center on the day prior to surgery. Exclusion criteria comprised any history of neurological or psychiatric diseases, any signs of delayed development in the child, isolation required because of multiresistant bacteria, inability of the parents to speak, read or understand the German language, as well as concurrent enrollment in another study.

The a priori primary outcome was ED assessed during stay in the recovery room with the Pediatric Assessment of Emergence Delirium (PAED) Score [15]. Values of at least 10 were considered as ED.

Secondary outcomes related to frontal EEG recordings were depth of anaesthesia and burst suppression duration.

Oral premedication with midazolam was administered in all children. Children were anaesthetised with either propofol or sevoflurane as decided by the anaesthesiologist in charge. Standard monitoring included non-invasive blood pressure, electrocardiogram and pulse oximetry. Patients received i.v. propofol (assessed as mg kg-1 body weight) or mask induction / maintenance with sevoflurane (assessed as endtidal concentrations et Vol%). Dosage of sevoflurane and propofol was given according to clinical needs and chosen by the anaesthesiologist in charge. Remifentanil was administered as analgesic agent during the induction period in all children, according to clinical needs. If muscle relaxation was needed, cis-atracurium was administered adapted to body weight. Before the end of surgery children received metamizol or paracetamol and / or piritramid for analgesia. Some children also received regional anaesthesia. The complete anaesthetic procedure and medication were outside of the scope of this study.

### ED assessment

ED was assessed according to the PAED Score. From admission to the post-anaesthesiological care unit until discharge the PAED score was determined 1 min after extubation, at arrival in the recovery room, 5 min, 10 min, 15 min, 30 min, 45 min, and 60 min after arrival and at discharge from the recovery room by a member of the study staff sitting next to the child. Values > 10 were considered as an emergence delirium [15]. The “Faces Legs Activity Cry Consolability Pain Scale” was used to determine pain events [16]. Richmond Agitation Sedation Scale score was used to assess the level of consciousness [17]. PAED score was only included in the analysis if the Richmond Agitation Sedation Scale score was above − 2 and if it was unlikely that pain was triggering agitated behavior. If inadequate behavior during the stay in the recovery room improved after pain-medication these periods of agitated behavior were not classified as ED. If a member of the study team was unable to take the PAED score in the recovery room the child was excluded from further evaluation.

### EEG recording and analysis

Bi-frontal EEGs were obtained with the Narcotrend Monitor (MT Monitor Technik, Bad Bramstedt, Germany). The EEG was recorded continuously from baseline before start of anaesthesia until the end of stay in the recovery room. After skin preparation with alcohol four electrodes (Ambu BlueSensor, Bad Nauheim Germany) were placed on the patients’ forehead at positions Fz, F7 and F8, with a reference electrode at Fp2. The impedances were kept below 8 kΩ, differences between electrodes were less than 2 kΩ. During the EEG recording event markers comprising “start of anaesthesia”, “intubation”, “operation” and “extubation” and “recovery room” were placed. “Start of anaesthesia” was defined as the time-point when the anaesthesiologist began to administer the anaesthetic agent, i.e., either sevoflurane, propofol or a mixture of both. “Intubation” was defined as the time point, when the anaesthesiologist in charge began to intubate the child. “Operation” indicated a time point within 15 min to 30 min after intubation of stable surgery and anaesthesia, without severe pain events or intraoperative bolus application of propofol. “Extubation” was defined as the time point, at which the anaesthesiologist in charge extubated the child.

Raw EEG data were recorded with a high pass filter of 0.5 Hz and a low pass filter of 45 Hz, sampling rate was 128 / sec. Visual EEG analysis (EEG viewer software: 50 μV–100 μV and 1 s/div) was performed from the time point of “start of anaesthesia” until “extubation”. The raw EEG was analysed by an expert (S. K., neurologist with specialization in clinical neurophysiology and EEG) blinded to the ED outcome, the medication patients received and further clinical data. The presence of Burst Suppression periods was validated by a second expert (C.P. pediatric neurologist with specialization in electroencephalography in children). Short periods (< 5 min within the total anaesthesia procedure) with artifacts (muscle, eyelid, and electricity) were excluded from any further analysis. If persistent artifacts or repeating artifacts were seen, these EEGs were excluded from further analysis.

Burst Suppression periods were assessed by visual inspection of the raw EEG (EEG viewer software: 50 μV and 1 s/div). Burst Suppression segments were included if duration of isoelectric line exceeded 0.5 s. Duration of intraoperative Burst Suppression was calculated from start of the first isoelectric line segment until the end of the last isoelectric line. Isoelectric line is classified as an EEG activity below 5 μV [18]. We calculated the duration of the isoelectric line activity by distracting the time duration of the intermittent burst activity. Duration of the isoelectric line is the sum of all periods of isoelectric line present in the raw EEG. Additionally, we calculated the Burst Suppression strength by dividing total isoelectric line duration by Burst Suppression duration. The Burst Suppression strength indicates the fraction of the isoelectric line within the Burst Suppression pattern, a prolonged duration of an isoelectric line will show a Burst Suppression strength value tending towards 1, which indicates a deeper stage of coma or a deeper level of anaesthesia.

### Statistical analysis

The present study was designed as a prospective, observational study. Statistical analysis was performed using SPSS, version 25, copyright SPSS, Inc., Chicago, IL 60606, USA. Data are presented as means + SD, in case of unbalanced data distribution as medians (IQR 25/75) or as frequencies (%). For nominal data statistical analysis was performed with the Chi-square test from Pearson. Numerical data were analysed using the Mann-Whitney-U test for non-parametric data. To determine the impact of age, anaesthetic medication, Burst Suppression and depth of anaesthesia on the incidence of ED we performed a univariate logistic regression. Odds Ratio with 95% confidence intervals and corresponding *p*-values were calculated for each risk factor. The Spearman Rho correlation test was used to analyse the correlation between age (in months) and Burst Suppression duration, isoelectric line duration and Burst Suppression strength. Statistical significance was assumed at *p* < 0.05.

The statistical analysis plan was made prior to data assessment. To calculate the sample size needed we postulated an ED incidence of 10.5% (from a pilot study in our department 2013 (NCT02358278), resulting in a 10.5% incidence of ED in children aged 0–14 years) and an increased risk to develop ED with an increase in depth of anaesthesia and the occurrence of Burst Suppression periods. Four hundred seventy patients were initially planned to be included in this study. An interim analysis was planned (after approximately 1/3–1/2 of the total planned sample size) with recalculation of the initial sample size calculation to adopt the study procedure, if the initial assumptions differed strongly. At the interim analysis the ED incidence rate was distinctly higher, which is most likely related to the fact that we mainly included younger patients in our actual study compared to the pilot study 2013 in our department. We re-ran the sample size calculation taking into account the new incidence rate of 41%. Initially, the study was planned for an odds ratio of 1.6 with an ED incidence rate of 10.5% and a R2 of the other covariates of 0.2 resulting into a sample size of 470 children with a power of 80%. Considering the new incidence rate of 41% and without changes of the other parameters we achieve a power of 80% with 97 children and an odds ratio of 2.1.

## Results

A total of 412 children were screened at the preoperative evaluation center. One hundred eighty-nine children have been included in this prospective observational study. The study suffers a high dropout rate based on different reasons. Especially EEG recordings were incomplete because of agitated behaviour in children during anaesthesia induction making it impossible to applicate EEG electrodes before anaesthesia induction without increasing the discomfort or reduce the safety for the young children. Finally, intraoperative EEG data analysis could be performed from “start of anaesthesia” until “extubation” in 97 children (Additional file 1: Figure S1, Flow chart).

Of these 97 children 40 (41%) children developed Emergence delirium (ED group) and 57 (59%) did not (Non-ED group). Patients’ characteristics are presented in Table 1. ED patients were significantly younger of age (*P* = 0.042).

**Table 1:** Patients’ characteristics for children with emergence delirium and children without emergence delirium. Values are median (IQR range), number (propotion) or mean + SD

	Non-ED Group (*n* = 57)	ED Group (*n* = 40)	*P* value
Age [months]	58 (15.75 / 78)	22 (13 / 60)	0.042
Weight (kg)	17 (9.7 / 22)	11 (9.3 / 22)	0.142
Height (cm)	107.5 (80 / 131)	86 (81.5 / 119.5)	0.255
ASA Score (I / II / III) (%)^a^	46 / 11 / 0 (81 / 19 / 0)	32 / 7 / 1 (80 / 17,5 / 2,5)	0.480
Gender male / female (%)	35 / 22 (61 / 39)	25 / 15 (62,5 / 37,5)	0.913
Procedures (%)			
Cleft-lip-palate; Oral / neck surgery	6 (11)	13 (33)	0.102
Inguinal hernia / Circumcision / Orchidopexy / Cystoscopy	15 (26)	8 (20)	
Otorhinolaryngology surgery	7 (12)	4 (10)	
Intraabdominal surgery / long procedures (> 60 min)	14 (25)	9 (23)	
Limb surgery / short procedures (< 60 min)	15 (26)	6 (15)	
Midazolam premedication (mg/kg body weight)	0.64 + 0.17	0.67 + 0.18	0.180
Induction Agent Sevoflurane / Propofol / mixed (%)	12 / 23 / 22 (21 / 41 / 39)	9 / 13 / 18 (22,5 / 32,5 / 45)	0.724
Maintance Agent Sevoflurane / Propofol / mixed (%)	38 / 18 / 1 (67 / 31 / 2)	27 / 13 / 0 (67,5 / 32,5 / 0)	0.971
Anaesthesia duration (min)	102 + 71	146 + 113	0.159

Patient characteristics for children without emergence delirium (Non ED group) and children with ED (ED group). Age (*p* = 0.042) differed significantly between NonED group vs. ED group. (Chi-square Pearson and Mann-Whitney-U Test) ^a^*ASA* American Society of Anesthesiologists

51.5% (*n* = 50) of all children developed Burst Suppression periods during the anaesthesia procedure, while the remaining 48.5% (*n* = 47) did not. Burst Suppression started approximately within ~ 4 min after “start of anaesthesia” (223 s (IQR 123 to 412), and ended ~ 11 min after “start of anaesthesia” (676 s (IQR 417 to 1413)). Burst Suppression duration was 294 + 502 s and isoelectric line duration was median 179 + 375 s.

Occurrence and duration of Burst Suppression, duration of isoelectric line, as well as Burst Suppression strength was not significantly different between ED patients and Non ED patients (Table 2).

**Table 2:** EEG suppression for children with Emergence Delirium and children without emergence delirium. Values are number (propotion) or mean + SD

	Non-ED Group (*n* = 57)	ED Group (*n* = 40)	*P* value
Burst Suppression yes / no (%)	28 / 29 (49 / 51)	22 / 18 (55 / 45)	0.569
Burst Suppression duration (sec)	318 + 531	261 + 462	0.984
Isoelectric line duration (sec)	192 + 407	159 + 328	0.889
Burst Suppression strength	0.55 + 0.32	.59 + 0.31	0.762

Comparing EEG suppression during anaesthesia for children with Emergence delirium (ED group) and children without Emergence delirium (Non ED group). (Chi-square Pearson and Mann-Whitney-U Test). Burst Suppression strength is calculated by dividing isoelectric line duration over Burst Suppression duration.

Calculation of univariate logistic regression for confounders considered as risk factors triggering ED [age (months), anaesthetic agent given at induction / maintenance (sevoflurane vs. propofol), anaesthesia duration (min), and EEG suppression (occurrence, duration (sec), strength] only age (*P* = 0.046) and anaesthesia duration (*P* = 0.025) showed a significant association (Table 3).

**Table 3:** Confounders considered risk factors for emergence delirium in children

Confounders	Odds ratio	95% CI lower limit	95% CI upper limit	*P* Value
Age (months)	0.986	0.973	1.000	0.046
Anaesthesia Induction (Sevoflurane / Propofol / mix of both)	0.691	0.275	1.737	0.432
Anaesthesia Maintenance (Sevoflurane / Propofol)	0.699	0.427	2.420	0.971
Concentration of Midazolam (mg/kg body weight)	2.886	0.253	32.867	0.393
Concentration of Remifentanil (μg/kg body weight / min)	0.465	0	798.215	0.840
Anaesthesia duration (min)	1.005	1.001	1.010	0.025
Burst suppression occurrence (yes / no)	1.266	0.563	2.848	0.569
Burst suppression duration (sec)	1	0.999	1.001	0.583
Burst Suppression strength	0.9	0.721	1.122	0.349

Univariate logistic regression accounting for confounders considered risk factors triggering emergence delirium after general anaesthesia in children. Only age (months) and anaesthesia duration (min) showed a significant association

To rule out the risk that younger age has biased our results we recalculated the impact of Burst Suppression duration (sec), isoelectric line duration (sec) and Burst Suppression strength on ED in a pair – wise age matched group (matching rules: age should not differ more than 2 months in children younger than 60 months and not more than 6 months in children being older than 60 month). But again we did not find an impact of Burst Suppression duration, isoelectric line duration and Burst Suppression strength on the risk to develop ED (Additional file 2: Table S1).

Burst suppression periods occurred more frequently in children receiving a mixed induction of anaesthesia with sevoflurane and propofol, compared with children receiving propofol or sevoflurane alone (Burst suppression occurrence: sevoflurane induction 24%; propofol induction 46%, mixed induction 68%; R^2^ 0.323, *P* = 0.001) (Additional file 3: Figure S2). Duration of Burst Suppression, as well as duration of isoelectric line were related to the anaesthetic agents given for induction and maintenance (Additional file 4: Figure S3).

We did not find a correlation between Burst Suppression duration (sec) and isoelectric line duration (sec) and age (months), concentration of midazolam (mg kg^− 1^ body weight) premedication or concentration of remifentanil (μg kg^− 1^ body weight min^− 1^) intraoperatively. However, Burst Suppression strength showed a high correlation with age (in months), a tendency to display a longer fraction of isoelectric line within the Burst Suppression pattern in younger children (Spearman-Rho Correlation coefficient 0.528, *p* < 0.0001; Additional file 5: Table S2 and Additional file 6: Figure S4).

## Discussion

We were able to show that occurrence and duration of Burst Suppression in children aged 0.5–8 years is not associated with the incidence of ED. Overall, Burst Suppression occurred in about 52% of all children aged 0.5–8 years, appearing mainly within the first ~ 4 min after application of the anaesthetic agents at start of anaesthesia.

Delirium is the most frequent brain dysfunction seen after anaesthesia procedures, mainly occurring in elderly patients and preschool children [19]. In elderly patients it has been shown that periods of Burst Suppression and a deep index level of anaesthesia are related to postoperative delirium [9–12], however, in two studies analyzing the index level of anaesthesia in children a relationship with the occurrence of ED was not found [13, 14].

Faulk and colleagues examined 400 children aged 1–12 years scheduled for dental procedures. Deep hypnosis was defined as a level of BIS index reading of less than “45”. They did not find a correlation between deep hypnosis and the occurrence of ED [13].

Frederick and colleagues undertook a randomized controlled trial including 40 children aged 2–8 years, randomized in a low-normal group (BIS index values 40–45) versus high-normal group (BIS index values 55–60). They found no significant effect of deep versus light anaesthesia on the incidence of ED [14].

In both studies the underlying hypothesis that deep index levels of anaesthesia in children might be related to ED, was not confirmed. This is in line with our data, as we found that the occurrence and duration of Burst Suppression activity was not related to ED in children. Moreover, we extended this conclusion, since our results are based on raw EEG data analysis, which is known to be more reliable compared to Burst Suppression ratios indicated within the processed EEG [20]. Burst suppression segments in the EEG are characterized by an isoelectric line interrupted by high-voltage EEG bursts, indicating a very deep state of unconsciousness with a marked reduction in brain metabolism [21]. The occurrence of Burst Suppression during general anaesthesia in elderly patients is positively correlated to the incidence of postoperative delirium, hence one might propose that elderly patients struggle to restore the preoperative brain metabolism level. In contrast, in children neuronal hyperexcitability – as seen by occurrence of interictal, epileptiform discharges during anaesthesia induction or increased frontal connectivity during emergence of general anaesthesia – has been related to ED [7, 8]. These striking differences in EEG activity related to delirium in children compared with elderly patients extends into its clinical presentation, where delirious children present with crying, agitation, disorientation and altered response to their surroundings, whereas elderly patients present primarily with a hypoactive form of delirium.

Additionally, we found that preschool children display Burst Suppression periods to a lower extent during general anaesthesia procedures (52%) compared with older patients (aged 62 + 14 years; 89%), however younger adults seem to have the lowest risk (below ~ 25%) to show Burst Suppression activity during general anaesthesia [12, 22].

### Limitations

In our study children receiving a mask induction with sevoflurane were significantly younger compared with children receiving an i.v. induction with propofol, due to standard operating procedure in our clinic to try to avoid children’s discomfort during anaesthesia induction. Since younger age as well as sevoflurane anaesthesia have been described to cause ED, this may have biased our results. However, the Non-ED versus ED-group did not show a significant difference between anaesthetic agent used for induction or maintenance and an age-matched sub-group analysis did also not reveal a correlation between Burst Suppression duration and ED.

## Conclusion

Intraoperative Burst Suppression activity in the EEG is not associated with ED in young children. Burst Suppression activity is a characteristic EEG feature of a pathological, profoundly inactivated brain but despite its pathological character this does not render to the occurrence of ED in children.

## Additional files

Additional file 1 : **Figure S1** Flow diagram. Showing the study flow chart. (DOCX 38 kb)

Additional file 2 : **Table S1** Age-matched subgroup analysis. The table presents in an age-matched subgroup analysis Burst Suppression duration, isoelectric line duration and Burst Suppression strength for children with ED versus Non ED group, to rule out any influence of age on our primary result. (DOCX 20 kb)

Additional file 3 : **Figure S2** Burst Suppression occurrence related to the anaesthetic agent at induction. Burst suppression periods occurred more frequently in children receiving a mixed induction of anaesthesia with sevoflurane and propofol, compared with children receiving propofol or sevoflurane alone. (DOCX 90 kb)

Additional file 4 : **Figure S3** Duration of Burst Suppression activity in the EEG related to the anaesthetic agent used for induction and maintenance. Box plot diagram showing the differences of Burst Suppression duration related to the anaesthetic agent used for anaesthesia induction and maintenance. (DOCX 29 kb)

Additional file 5 : **Table S2** Correlation between Burst Suppression duration, isoelectric line duration, Burst Suppression strength and age (month). Spearman Rho correlation analysis showing no correlation between Burst Suppression duration, isoelectric line duration and age (month), but a significant correlation between age (month) and Burst Suppression strength, indicating that younger children show prolonged periods of isoelectric line within a Burst Suppression pattern. (DOCX 19 kb)

Additional file 6 : **Figure S4** Burst Suppression strength related to age (month). Scatter polt indicating the tendency to display a longer fraction of isoelectric line within the Burst Suppression pattern in younger children. (DOCX 40 kb)

## Data Availability

The datasets used and/or analysed during the current study are available from the corresponding author on reasonable request.

## Abbreviations

ED: Emergence delirium
EEG: Electroencephalography / Electroencephalogramm
