# Supplementary material for: Emergence delirium in children is not related to intraoperative burst suppression – prospective, observational electrography study
Source: BMC Anesthesiol. 2019 Aug 8;19:146. doi: 10.1186/s12871-019-0819-2 (PMC6688308; doi:10.1186/s12871-019-0819-2)
Supplement: Supplementary file 1 — : Figure S1 Flow diagram. Showing the study flow chart. (DOCX 38 kb) [file 12871_2019_819_MOESM1_ESM.docx]

**Figure s1 Flow diagram**

Study drop out (n=34)

- surgeries were delayed / cancelled (n=17)
- primary outcome evaluation was missed due to organizational problems/admission intensive care unit (n=10)
- parents withdraw their consent (n=4)
- included in an interventional study, not obvious at inclusion (n=1)
- organizational problems (n=1)
- neurological disease, not obvious at inclusion (n=1)

EEG drop out (n=58)

- had no intraoperative EEG recording (n=54)
- EEG analysis not possible due to artifacts intraoperative (n=4)

Included in analysis (n=97)

Non Emergence delirium (n=57)

Emergence delirium (n=40)

Excluded (n=223)

- Not meeting inclusion criteria (n=104)
- Declined to participate (n=112)
- Other reasons (n=7)

Included in study (n=189)

Assessed for eligibility (n=412)
