# Supplementary material for: Emergence delirium in children is not related to intraoperative burst suppression – prospective, observational electrography study
Source: BMC Anesthesiol. 2019 Aug 8;19:146. doi: 10.1186/s12871-019-0819-2 (PMC6688308; doi:10.1186/s12871-019-0819-2)
Supplement: Supplementary file 2 — : Table S1 Age-matched subgroup analysis. The table presents in an age-matched subgroup analysis Burst Suppression duration, isoelectric line duration and Burst Suppression strength for children with ED versus Non ED group, to rule out any influence of age on our primary result. (DOCX 20 kb) [file 12871_2019_819_MOESM2_ESM.docx]

**Table s1** Age-matched subgroup analysis ED versus Non ED group for age and Burst Suppression duration, isoelectric line duration, Burst Suppression strength.

| **Age-matched group** | Emergence Delirium | N | Mean | SD | p-value |
| --- | --- | --- | --- | --- | --- |
| age (month) | noED | 35 | 40.37 | 31.94 |  |
|  | ED | 35 | 40.49 | 32.24 | 0.995 |
| BS duration (sec) | noED | 35 | 355.43 | 622.08 |  |
|  | ED | 35 | 260.14 | 456.86 | 0.877 |
| isoelectric line duration (sec) | noED | 35 | 255.71 | 493.89 |  |
|  | ED | 35 | 156.74 | 330.38 | 0.970 |
| BS strength | noED | 35 | 0.677 | 0.29 |  |
|  | ED | 35 | 0.57 | 0.32 | 0.198 |

Impact of Burst Suppression duration, isoelectric line duration and Burst Suppression strength on ED in an age-matched subgroup analysis. Accepted matching rules: 6month differences for children older than 60month and 2month difference for children younger than 60month for each pair. Based on these matching terms we could include 35 patients in each group. We did not find a significant difference in Burst Suppression duration (sec), isoelectric line duration (sec) and Burst Suppression strength between ED and Non-ED group (Mann-Whitney-U test). BS = Burst Suppression.
