# Supplementary material for: Emergence delirium in children is not related to intraoperative burst suppression – prospective, observational electrography study
Source: BMC Anesthesiol. 2019 Aug 8;19:146. doi: 10.1186/s12871-019-0819-2 (PMC6688308; doi:10.1186/s12871-019-0819-2)
Supplement: Supplementary file 3 — : Figure S2 Burst Suppression occurrence related to the anaesthetic agent at induction. Burst suppression periods occurred more frequently in children receiving a mixed induction of anaesthesia with sevoflurane and propofol, compared with children receiving propofol or sevoflurane alone. (DOCX 90 kb) [file 12871_2019_819_MOESM3_ESM.docx]

**Figure s2** Burst Suppression occurrence related to the anaesthetic agent at induction


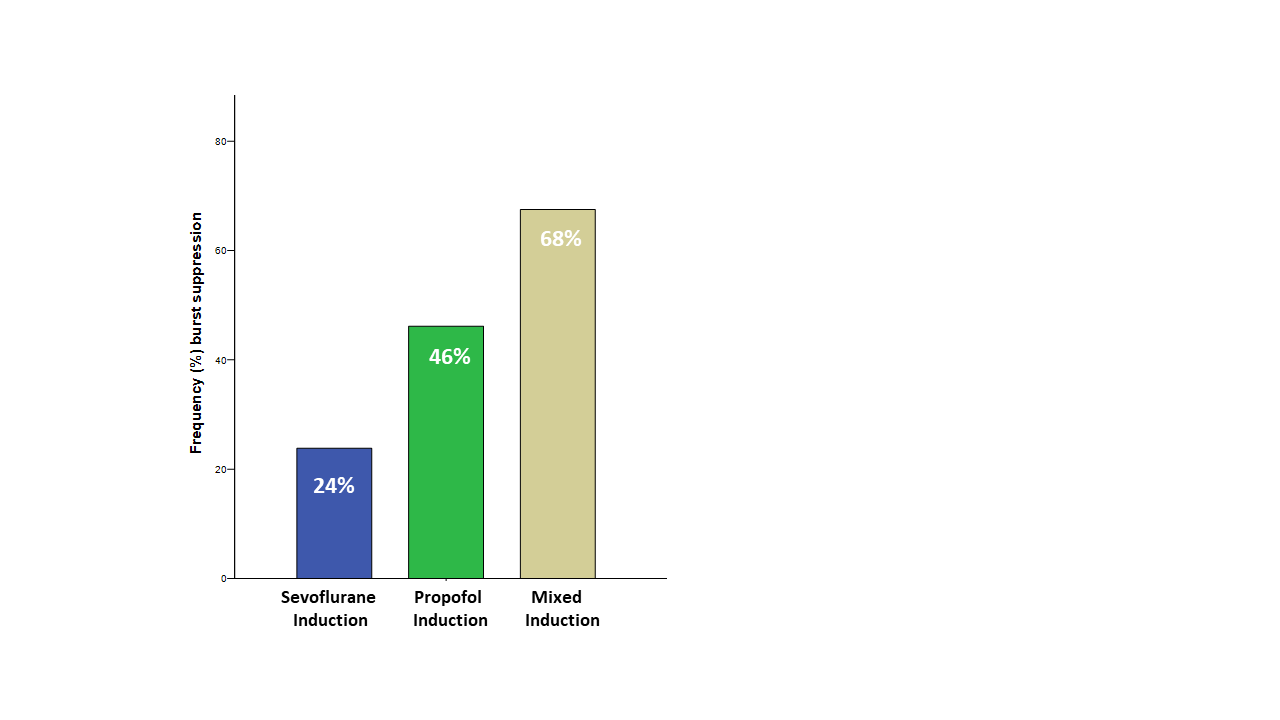


Burst suppression periods occurred more frequently in children receiving a mixed induction of anaesthesia with sevoflurane and propofol, compared with children receiving propofol or sevoflurane alone (Burst suppression occurrence: sevoflurane induction 24%; propofol induction 46%, mixed induction 68%; R² 0.323, P = 0.001).
