# Supplementary material for: Emergence delirium in children is not related to intraoperative burst suppression – prospective, observational electrography study
Source: BMC Anesthesiol. 2019 Aug 8;19:146. doi: 10.1186/s12871-019-0819-2 (PMC6688308; doi:10.1186/s12871-019-0819-2)
Supplement: Supplementary file 4 — : Figure S3 Duration of Burst Suppression activity in the EEG related to the anaesthetic agent used for induction and maintenance. Box plot diagram showing the differences of Burst Suppression duration related to the anaesthetic agent used for anaesthesia induction and maintenance. (DOCX 29 kb) [file 12871_2019_819_MOESM4_ESM.docx]

**Figure s3** Duration of Burst Suppression activity in the EEG related to the anaesthetic agent used for induction and maintenance.

Duration of Burst Suppression, as well as duration of isoelectric line were related to the anaesthetic agents given for induction (*Burst suppression duration:* sevoflurane induction 0 sec (IQR 0 to 113), propofol induction 0 sec (IQR 0 to 230); mixed induction 180 sec (IQR 0 to 824); P = 0.003; *Isoelectric line duration:* sevoflurane induction 0 sec (IQR 0 to 113); propofol induction 0 sec (IQR 0 to 43); mixed induction 84 sec (IQR 0 to 385); P = 0.002) and for anaesthesia maintenance (*Burst suppression duration:* sevoflurane 0 sec (IQR 0 to 229); propofol 300 sec (IQR 0 to 600); P = 0.051; *isoelectric line duration*: sevoflurane 0 sec (IQR 0 to 68); propofol 83 sec (IQR 0 to 373); P = 0.055).
