# Supplementary material for: Emergence delirium in children is not related to intraoperative burst suppression – prospective, observational electrography study
Source: BMC Anesthesiol. 2019 Aug 8;19:146. doi: 10.1186/s12871-019-0819-2 (PMC6688308; doi:10.1186/s12871-019-0819-2)
Supplement: Supplementary file 5 — : Table S2 Correlation between Burst Suppression duration, isoelectric line duration, Burst Suppression strength and age (month). Spearman Rho correlation analysis showing no correlation between Burst Suppression duration, isoelectric line duration and age (month), but a significant correlation between age (month) and Burst Suppression strength, indicating that younger children show prolonged periods of isoelectric line within a Burst Suppression pattern. (DOCX 19 kb) [file 12871_2019_819_MOESM5_ESM.docx]

**Table s2** Correlation between Burst Suppression duration, isoelectric line duration, Burst Suppression strength and age (month).

|  | | | BS duration (sec) | isoelectric line duration (sec) | BS - strength |
| --- | --- | --- | --- | --- | --- |
|  | age (month) | Correlation coefficient | ,053 | -,002 | ,528^**^ |
|  |  | Sig. (2-tailed) | ,604 | ,982 | ,000 |

We did not find a significant correlation for Burst Suppression duration (sec) and isoelectric line duration (sec) and age (month) but a significant correlation between Burst-Suppression strength and age (Spearman Rho correlation analysis).
