# Supplementary material for: Emergence delirium in children is not related to intraoperative burst suppression – prospective, observational electrography study
Source: BMC Anesthesiol. 2019 Aug 8;19:146. doi: 10.1186/s12871-019-0819-2 (PMC6688308; doi:10.1186/s12871-019-0819-2)
Supplement: Supplementary file 6 — : Figure S4 Burst Suppression strength related to age (month). Scatter polt indicating the tendency to display a longer fraction of isoelectric line within the Burst Suppression pattern in younger children. (DOCX 40 kb) [file 12871_2019_819_MOESM6_ESM.docx]

**Figure s4** Burst Suppression strength related to age (month).


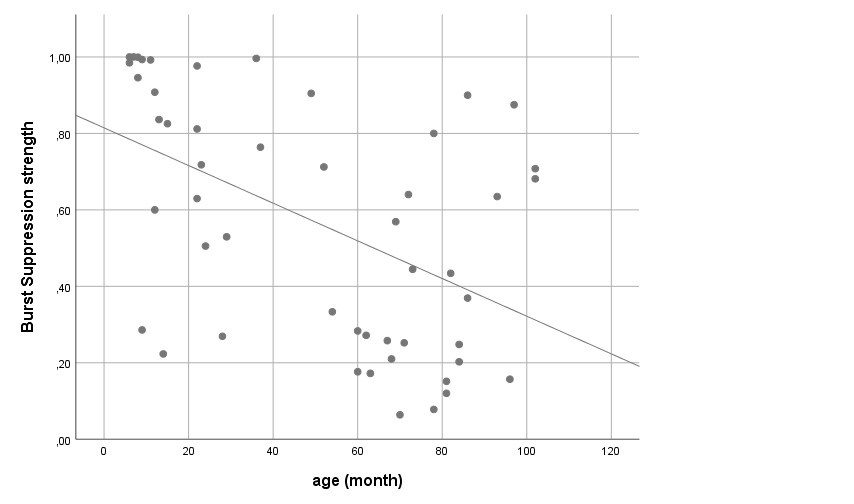


Burst Suppression strength shows a high correlation with age (month), indicating a tendency to display a longer fraction of isoelectric line within the Burst Suppression pattern in younger children.
